# Monoclonal Antibodies and Toxins—A Perspective on Function and Isotype

**DOI:** 10.3390/toxins4060430

**Published:** 2012-06-11

**Authors:** Siu-Kei Chow, Arturo Casadevall

**Affiliations:** 1 Department of Microbiology and Immunology, Albert Einstein College of Medicine, 1300 Morris Park Ave, Bronx, NY 10461, USA; Email: siukei.chow@phd.einstein.yu.edu; 2 Division of Infectious Diseases of the Department of Medicine, Albert Einstein College of Medicine, 1300 Morris Park Ave, Bronx, NY 10461, USA

**Keywords:** antibody, neutralization, protection, clearance, vaccine, therapeutics, isotype, animal model, *in vivo*, disease enhancement

## Abstract

Antibody therapy remains the only effective treatment for toxin-mediated diseases. The development of hybridoma technology has allowed the isolation of monoclonal antibodies (mAbs) with high specificity and defined properties, and numerous mAbs have been purified and characterized for their protective efficacy against different toxins. This review summarizes the mAb studies for 6 toxins—Shiga toxin, pertussis toxin, anthrax toxin, ricin toxin, botulinum toxin, and Staphylococcal enterotoxin B (SEB)—and analyzes the prevalence of mAb functions and their isotypes. Here we show that most toxin-binding mAbs resulted from immunization are non-protective and that mAbs with potential therapeutic use are preferably characterized. Various common practices and caveats of protection studies are discussed, with the goal of providing insights for the design of future research on antibody-toxin interactions.

## 1. Introduction

Humoral immunity against bacterial toxins has been studied for over a century. This research journey started when Behring and Kitasato established the existence of humoral immunity by showing that passive transfer of antibodies from the blood of immunized animals could protect non-immune animals against diphtheria [1]. Serum therapy was commonly used to treat infectious diseases until the discovery of sulfonamide in the 1930s [2]. Today serum therapy remains the only prophylactic and therapeutic option against many toxin-mediated and viral diseases. Toxins are excellent target antigens for antibodies, as they are usually structurally distinct from the self-antigens expressed by the host cells. In contrast, anti-infective drugs are usually not selective enough to differentiate pathogenic strains from non-pathogenic flora and thereby have the potential to cause a long-term disruption on the flora dynamics [3]. To date specific antibody is the only compound that can neutralize toxin and they are attractive for therapeutic development because antimicrobial drugs can kill the microbes but does not eradicate pre-formed toxins.

The development of hybridoma technology in 1975 allowed, for the first time, the functional and structural analysis of individual immunoglobulin molecules [4]. Individual antibody molecules can now be isolated in monoclonal preparations and produced in an unlimited supply. The availability of monoclonal antibodies (mAbs) with defined specificity and expressing a single isotype significantly reduces its toxicity relative to immune serum [5], despite the fact that non-human mAbs used directly as therapeutics against human diseases are still immunogenic [6]. The high purity of the preparation also enhances the biological activity per mass of protein [7], since all immunoglobulins are specific for the target toxin. This is particularly important for the development of prophylactic and therapeutic antibodies against infectious and toxin-mediated diseases [8]. In addition to the neutralizing activity that is mediated by the binding of the variable region to toxin, the Fc region of mAb mediates various effector functions including antibody-dependent cell-mediated cytotoxicity (ADCC), complement activation, and opsonization [9]. The combined potential of an immunoglobulin molecule for microbe eradication, toxin neutralization, and the enhancement of host immune system make mAbs attractive candidates for the next generation of magic bullet against infectious diseases.

The toxin neutralizing efficacy of an antibody molecule is usually defined by the activity of its monoclonal preparation. In general, a mAb can be classified as protective, indifferent, or disease-enhancing depending on how it modifies the course of infection or toxemia measured by various types of protection assays. Protective mAb benefits the host primarily by neutralizing the toxin, while disease-enhancing mAb intensifies its toxicity. Indifferent mAb binds to the toxin with no apparent effect on toxicity and host damage. This paper reviews the literature on mAbs to six toxins listed on Pubmed and analyzes the prevalence of the various functional types and isotypes. All the mAbs described result from immunization with whole toxin, toxin component/subunit, or clinical vaccine. Uncharacterized mAbs were not included in our analysis. Only those studies that described the first appearance of a particular mAb are listed—the follow up studies about the chimerization or humanization of a previously reported mAb will not be discussed in this review. In addition, we acknowledge that a significant number of toxin-binding fragment antigen binding (Fab) and single-chain variable fragment (scFv) have been generated as alternative forms of antibody-derived reagents that possess great potential in prophylaxis and therapeutics [10,11,12], but this paper will focus only on mAbs that originate from hybridoma cells. 

## 2. Selective Characterization of mAbs

**Figure 1:**
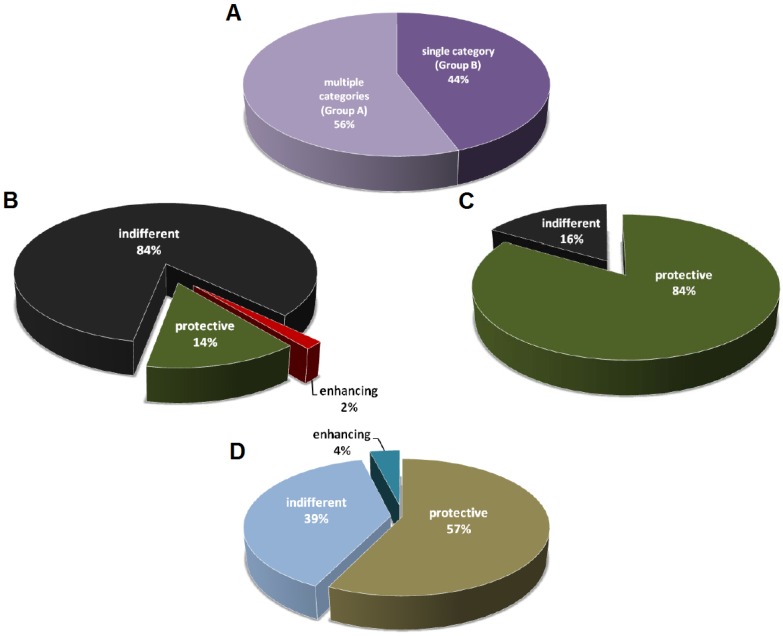
Distribution of mAbs classified as protective, indifferent, or disease-enhancing. (**A**) mAb studies divided into two groups. Group A consists of studies that describe multiple categories of mAbs (among protective, indifferent, and disease-enhancing). Group B consists of studies that describe only a single category of mAbs. *n* = 97 studies; (**B**) Distribution of mAbs with different activities from Group A studies. *n* = 2559 mAbs from 54 studies; (**C**) Distribution of mAbs with different activities from Group B studies. *n* = 111 mAbs from 43 studies; (**D**) Distribution of studies on protective, indifferent, or disease-enhancing mAbs.

The mAb studies listed in Table 1 are divided into two groups, with the first one (Group A) describing two or more categories of mAbs (among protective, indifferent, and disease-enhancing mAbs), and the second (Group B) describing a single category of mAbs (Figure 1A). In Group A 14% of the mAbs are protective, 2% are disease-enhancing, and the remainders are indifferent with no measurable effect on the host (Figure 1B). In contrast, for the studies that described only one category of mAbs, 84% of which are protective against toxins (Figure 1C). Humoral responses produce complex polyclonal mixtures with different specificities, isotypes, and affinities, and it is possible that the antibodies reported in Group A are more representative of natural antibody responses from immunization. The fact that a high percentage of the mAbs described in Group B is protective suggests that these studies are skewed to report primarily mAbs with prophylactic or therapeutic potential. A positive result publication bias could have led to significantly fewer publications for those studies that generated non-productive or indifferent mAbs. Consequently, the indifferent mAbs with no apparent benefits to the host are usually suggested as potential diagnostic or research tools. Not surprisingly, given the lack of therapeutic potential, disease-enhancing mAbs are never described on their own in any toxin-mAb studies. Disease-enhancing mAbs have been historically labeled as deleterious by-products of immunizations, while some of them are shown to enhance toxicity by increasing the activation rate of toxin components and through the interaction with the Fc region [13,14,15,16,17,18,19,20,21].

The field of antibody studies revolves around the discovery of novel and useful immunoglobulins against diseases, so it is not surprising that most effort is selectively given to characterize and report those mAbs with therapeutic potential (Figure 1D). While more than 20 mAbs have been developed and marketed as therapeutics against multiple types of cancers and inflammatory diseases, to date only one mAb is licensed for an infectious disease [22]. Palivizumab was approved by FDA in 1998 for the prevention of respiratory syncytial virus (RSV) infection in high-risk pediatric patients [23,24]. Despite of the very low approval rate of mAb against infectious diseases, research institutes and companies still devote large amount of time and resources in that area given that they remain the most reliable means to neutralize toxins. However, the effort in mAb discovery and development has always been spent on the candidates with the greatest potential given limited resources and keen competition [25], and this is evident in the fact that the majority of studies in Group B is focused on protective antibodies (Figure 1C).

**Table 1:** List of mAb studies against toxins.

Toxin	Authors	Reference	Binding sites/ domains/subunits	Protective	Indifferent	Enhancing	Uncharacterized	*In vivo* model
Shiga Toxin / Shiga-like Toxin	Griffin *et al*. (1983)	[26]	Stx	25 (IgG1)	63	-	-	-
	Donohue-Rolfe *et al*. (1984)	[27]	Stx	3 (IgG1)	-	-	-	-
	Strockbine *et al*. (1985)	[28]	SLT	3 (IgG1)	-	-	-	mouse (CD-1)
	Downes *et al*. (1988)	[29]	SLT-II	5 (IgG1)	-	-	-	-
	Perera *et al*. (1988)	[30]	SLT-II	5 (80% IgM and 20% IgG1)	-	-	-	-
	Donohue-Rolfe *et al*. (1989)	[31]	Stx, SLT-II	3 (66.6% IgG1 and 33.3% IgG2b)	2 (IgM)	-	-	-
	Padhye *et al*. (1989)	[32]	SLT-I, SLT-II	3 (66.6% IgG1 and 33.3% IgG2b)	11 (90.9% IgM and 9.1% IgG2b)	-	-	mouse
	Islam and Stimson (1990)	[33]	Stx	4 (IgG1)	-	-	-	mouse
	Qadri *et al*. (1993)	[34]	Stx	-	1 (IgM)	-	-	-
	Nakao *et al*. (1999)	[35]	SLT-II	1 (IgG1)	-	-	-	-
	Mukherjee *et al*. (2002)	[36]	SLT-I	5 (40% IgM and 60% IgG1)	5 (IgM)	-	-	mouse (Swiss Webster)
	Mukherjee *et al*. (2002)	[37]	SLT-II	8 (IgG1)	28 (96.4% IgG1 and 3.6% IgG3)	-	-	mouse (Swiss Webster) and gnotobiotic piglet
	Nakao *et al*. (2002)	[38]	SLT-I	1	-	-	-	-
	Tanikawa *et al*. (2008)	[39]	SLT-I	1 (IgG1)	1 (IgA)	-	-	-
Pertussis Toxin	Sato *et al*. (1984)	[40]	S1, S4	1 (IgG2a)	2 (IgG1)	-	-	mouse (Slc:ddY)
	Frank and Parker (1984)	[41]	S2	-	2 (IgG1)	-	-	mouse (CFW)
	Sato *et al*. (1987)	[42]	S2, S3	2 (IgG1)	-	-	-	-
	Kenimer *et al*. (1989)	[43]	S1, S4	3 (IgG1)	3 (IgG1)	-	-	-
	Lang *et al*. (1989)	[44]	S2, S3, S4	5 (40% IgG1 and 60% IgG2a)	8 (25% IgG1, 62.5% IgG2a and 12.5% IgG2b)	-	-	-
	Sato and Sato (1990)	[45]	S1, S2, S3, S2–3, S4	5 (IgG1)	15 (IgG1)	-	-	mouse
	Halperin *et al*. (1991)	[46]	S1, S3	3	-	-	-	mouse (CFW)
	Kenimer *et al*. (1991)	[47]	S1	-	2 (IgG1 and IgG3)	-	-	-
	Sato *et al*. (1991)	[48]	S2, S3, S2–3, S4, S5	2	10	-	-	mouse
	Walker *et al*. (1991)	[49]	S1, S2–3, S4	3 (IgG1)	-	-	-	-
	Zaccolo *et al*. (1992)	[50]	S3	1 (IgG1)	-	-	-	mouse (BALB/c)
	Lee *et al*. (1999)	[51]	Adenylate cyclase toxin	4 (75% IgG1 and 25% IgG2a)	8 (62.5% IgG1, 25% IgG2a, and 12.5% IgG2b)	-	-	-
	Pootong *et al*. (2007)	[52]	S1	1 (IgG1)	-	-	6	-
Anthrax Toxin	Little *et al*. (1988)	[53]	PA	2 (IgG1)	35 (17.1% IgM, 62.9% IgG1, 11.4% IgG2a, 8.6% IgG2b)	-	-	Fisher 344 rat
	Little *et al*. (1990)	[54]	LF	4 (IgG1)	59 (30.5% IgM, 40.7% IgG1, 22% IgG2a, 3.4% IgG3, 3.4% IgA)	-	-	Fisher 344 rat
	Little *et al*. (1994)	[55]	EF	1 (IgG1)	9 (88.9% IgG1, 11.1% IgG2a)	-	-	-
	Little *et al*. (1996)	[56]	PA63	2 (IgG1 and IgG2b)	-	-	-	Fisher 344 rat
	Zhao *et al*. (2003)	[57]	LF	1	-	-	-	mouse (nude)
	Belova *et al*. (2004)	[17]	PA	2	-	1	-	-
	Brossier *et al*. (2004)	[58]	PA	9	87	-	-	mouse (OF1)
	Kozel *et al*. (2004)	[59]	poly γ-D-glutamic acid	1 (IgG1)	-	-	4 (IgG3)	mouse (BALB/c)
	Mohamed *et al*. (2004)	[18]	PA	-	22	21 (IgG2a)	-	-
	Sawada-Hirai *et al*. (2004)	[60]	PA	3 (IgG1)	1 (IgG1)	-	-	Fisher 344 rat
	Lim *et al*. (2005)	[61]	LF	2 (IgG1)	-	-	-	Fisher 344 rat
	Chen *et al*. (2006)	[62]	PA, LF	2	4	-	-	Fisher 344 rat
	Gubbins *et al*. (2006)	[63]	PA	3 (IgG1)	8 (IgG1)	-	-	-
	Rivera *et al*. (2006)	[64]	PA	2 (IgG1 and IgG2b)	-	-	-	mouse (BALB/c)
	Vitale *et al*. (2006)	[65]	PA	1 (IgG1)	-	-	-	rabbit
	Albrecht *et al*. (2007)	[66]	PA, LF	2 (IgG1)	-	-	-	mouse (A/J)
	Kozel *et al*. (2007)	[67]	poly γ-D-glutamic acid	5 (IgG3)	1 (IgG1)	-	-	mouse (BALB/c)
	Staats *et al*. (2007)	[68]	PA, LF	2	2	-	-	mouse (BALB/c)
	Abboud *et al*. (2009)	[69]	PA	1 (IgG1)	3 (33.3% IgM, 66.7% IgG1)	-	-	mouse (BALB/c)
	Chen *et al*. (2009)	[70]	LF	2 (IgG1)	1	-	89	Fisher 344 rat
	Kelly-Cirino and Mantis (2009)	[71]	PA	1 (IgG1)	2 (IgG1 and IgG2a)	-	2	mouse (BALB/c)
	Rosenfeld *et al*. (2009)	[72]	PA	101	499	-	-	Fisher 344 rat, Hartley guinea pig
	Winterroth *et al*. (2010)	[73]	EF	1 (IgM)	5 (20% IgM, 80% IgG1)	-	-	mouse (A/JCr)
	Chen *et al*. (2011)	[74]	poly γ-D-glutamic acid	2 (IgG1 and IgG3)	-	-	3	mouse (BALB/c)
	Kulshreshtha and Bhatnagar (2011)	[75]	LF and EF	1 (IgG2b)	-	-	-	mouse (BALB/c)
	Leysath *et al*. (2011)	[76]	EF	3 (IgG1)	1 (IgG1)	-	78	mouse (BALB/cJ, C57BL/6J)
	Little *et al*. (2011)	[19]	PA	-	56	17	-	Fisher 344 rat
	vor dem Esche *et al*. (2011)	[77]	LF	1 (IgG1)	17	-	-	mouse (A/J)
	Chow *et al.* (manuscript in prep.)	-	PA	2 (IgG2a)	16 (IgG1)	6 (83.3% IgG1, 16.7% IgG2a)	-	mouse (BALB/c)
Ricin Toxin	Colombatti *et al*. (1986)	[20]	RT, RTA	3	4	1	13	-
	Colombatti *et al*. (1987)	[78]	RTB	1 (IgG2a)	-	-	-	-
	Chanh *et al*. (1993)	[79]	RT	1 (IgG1)	19	-	-	mouse (BALB/c)
	Lemley *et al*. (1994)	[80]	RTA	2 (IgG1)	-	-	-	mouse (BALB/c)
	Maddaloni *et al*. (2004)	[21]	RT, RTA, RTB	18	19	1 (IgG1)	-	mouse (CD-1)
	Dertzbaugh *et al*. (2005)	[81]	RT, RTA, RTB	6 (IgG1)	23	-	-	-
	Mantis *et al*. (2006)	[82]	RTA, RTB	4 (IgA)	-	-	20 (IgA)	-
	McGuinness and Mantis (2006)	[83]	RTB	1 (IgG1)	-	-	-	-
	Pelat *et al*. (2009)	[84]	RTA	1	18	-	-	-
	Neal *et al*. (2010)	[85]	RTA	1 (IgG1)	-	-	-	mouse (BALB/c)
	O'Hara *et al*. (2010)	[86]	RTA	24	394	-	-	mouse (BALB/c)
	Dai *et al*. (2011)	[87]	RTA	3 (IgG1)	14	-	-	mouse
	Prigent *et al*. (2011)	[88]	RTA, RTB	7	24	-	-	-
	Yermakova and Mantis (2011)	[89]	RTB	2	~100	-	-	mouse (BALB/c)
Botulinum Toxin	Oguma *et al*. (1982)	[90]	Type C1	2 (IgG1)	2 (IgG1)	-	-	mouse (ddY)
	Oguma *et al*. (1984)	[91]	Type C1, D	17	11	-	-	-
	Kozaki *et al*. (1986)	[92]	Type E	3 (IgG1)	3 (66.7% IgG1, 33.3% IgG2b)	-	-	-
	Ferreira *et al*. (1987)	[93]	Type A	-	1 (IgG1)	-	60	mouse (Swiss Webster)
	Simpson *et al*. (1990)	[94]	Type E	3	1	-	-	mouse
	Toratani *et al*. (1993)	[95]	ADP-ribosyltransferase C3	4 (IgG2b and IgG3)	-	-	-	-
	Cenci Di Bello *et al*. (1994)	[96]	Type A	-	7 (85.7% IgG1, 14.3% IgG2b)	-	-	mouse
	Noah *et al*. (1995)	[97]	Type B	-	4	-	-	mouse
	Amersdorfer *et al*. (1997)	[98]	Type A	2	3	-	-	-
	Brown *et al*. (1997)	[99]	Type F	3	23	-	-	mouse
	Pless *et al*. (2001)	[100]	Type A	33	455	-	-	mouse
	Wu *et al*. (2001)	[101]	Type A	2 (IgG1)	14	-	-	mouse (ICR)
	Kamata *et al*. (2002)	[102]	ADP-ribosyltransferase C3	1 (IgG1)	-	-	-	-
	Yang *et al*. (2004)	[103]	Type B	1 (IgG1)	-	-	-	mouse (ICR)
	Adekar *et al*. (2008)	[104]	Type A	1 (IgG1)	19	-	-	mouse (Swiss Webster)
	Adekar *et al*. (2008)	[105]	Type A	1 (IgG1)	-	-	-	mouse
	Adekar *et al*. (2008)	[106]	Type A	1 (IgM)	1 (IgM)	-	-	mouse (Swiss Webster)
	Zhou *et al*. (2009)	[107]	Type B	1	-	-	-	-
	Mazuet *et al*. (2010)	[108]	Type A	12 (66.7% IgG1, 33.3% IgG2a)	2 (IgG1)	-	-	mouse (Swiss Webster)
	Corbett *et al*. (2011)	[109]	Type A	1 (IgG1)	-	-	7	mouse (Swiss Webster)
	Montgomery *et al*. (2011)	[110]	Type C	-	1	-	-	mouse (CD-1)
Enterotoxin B (SEB)	Lin *et al*. (1988)	[111]	SEB	4 (25% IgM, 75% IgG1)	1	-	-	-
	Hamad *et al*. (1994)	[112]	SEB	2 (IgG1)	2 (IgG1 and IgG2b)	-	-	-
	Pang *et al*. (2000)	[113]	SEB	1 (IgG1)	-	-	-	-
	Tilahun *et al*. (2010)	[114]	SEB	21 (IgG1)	-	-	-	-
	Larkin *et al*. (2010)	[115]	SEB	4	6	-	-	mouse (BALB/c)
	Drozdowski *et al*. (2010)	[116]	SEB	3 (IgG1)	-	-	-	mouse (BALB/c)
	Varshnev *et al*. (2011)	[117]	SEB	3 (IgM, IgG1, IgA)	8 (62.5% IgG1, 37.5% IgG2a)	-	-	mouse (BALB/c)

**Figure 2:**
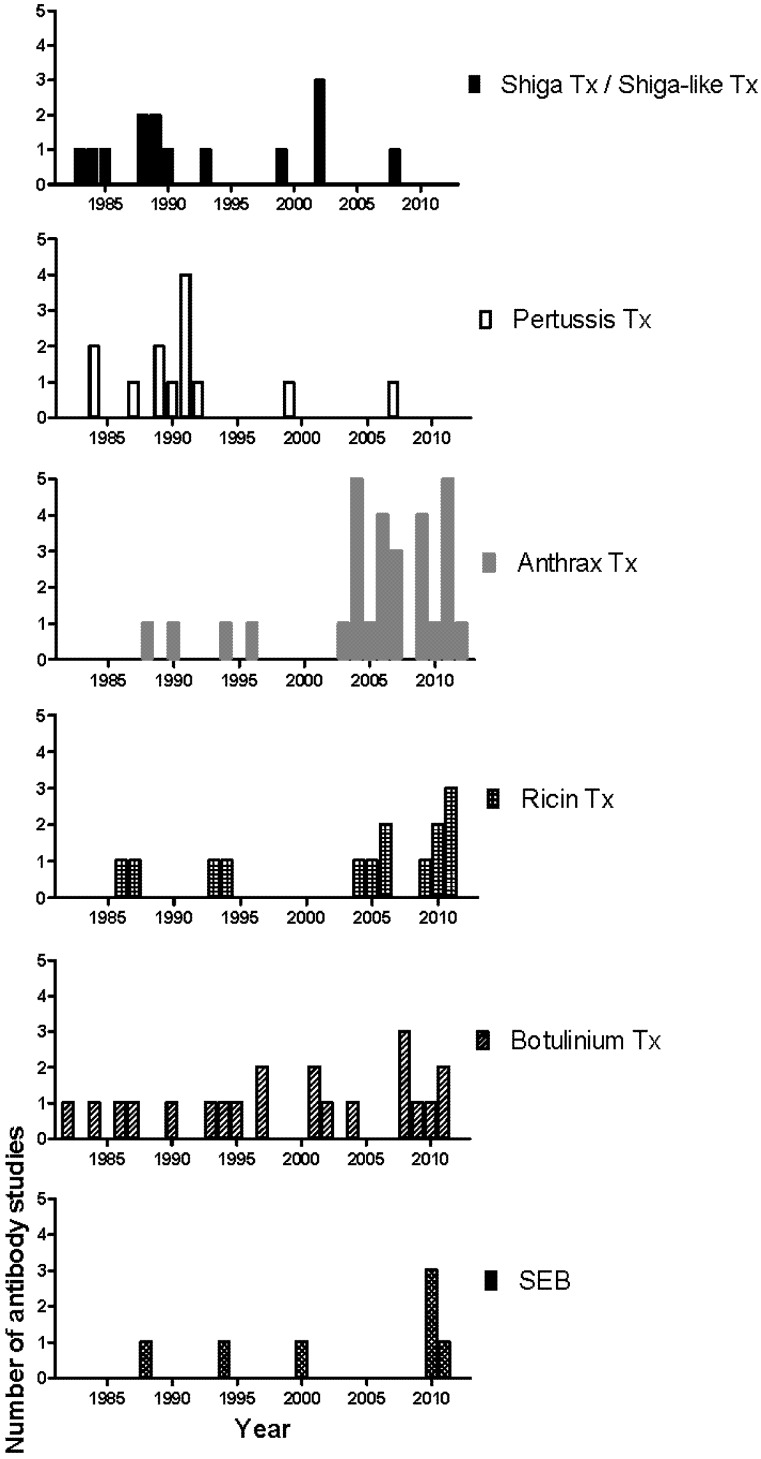
Chronological distribution of mAb studies for individual toxin.

The first phase of mAb development against most toxins began in the 80s and slowed down in the early 90s, a result from the wide application of the hybridoma technology and the successful isolation of toxins and their components (Figure 2). The second phase began after the increase of research spending on biological warfare toxins and the approval of the Project BioShield Act in 2004 that specifically aimed “to provide protections and countermeasures against chemical, radiological, or nuclear agents that may be in a terrorist attack against the United States by giving the National Institutes of Health (NIH) contracting flexibility, infrastructure improvements, and expediting the scientific peer review process, and streamlining the Food and Drug Administration (FDA) approval process of countermeasures” [118]. Consequently, the number of mAb studies on National Institute of Allergy and Infectious Diseases (NIAID) biodefense category A and B priority pathogens and their toxins, such as anthrax toxin, ricin toxin and Staphylococcus enterotoxin B (SEB) increased significantly (Figure 2) [118]. Many of these mAbs were further chimerized and humanized (data not shown). In 2009, Human Genome Sciences delivered 20,000 doses of Raxibacumab, a human IgG1 mAb as a treatment of inhalation anthrax, to the US Strategic National Stockpile, and an additional 45,000 doses were ordered later in the same year [119,120]. In contrast, for diseases with a less obvious threat profile and better vaccine/treatment such as those mediated by Shiga toxin, Shiga-like toxin, and pertussis toxin, the mAb development has slowed down since the late 90s (Figure 2). Progress in the generation of prophylactic and therapeutics mAbs against biodefense pathogens has recently been reviewed [121].

**Figure 3:**
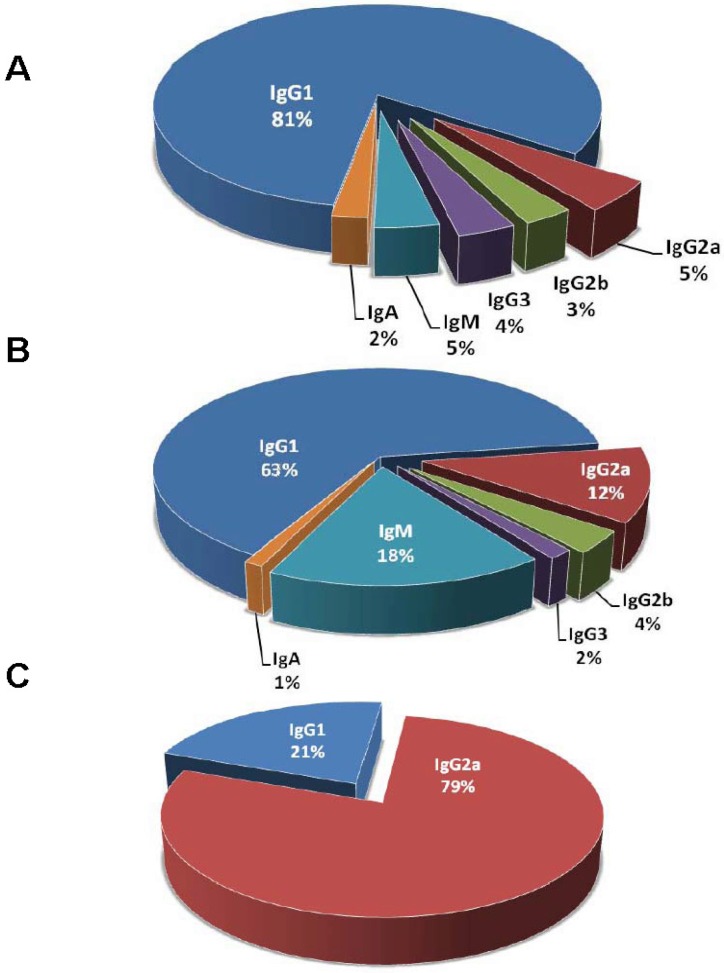
Distribution of mAb isotypes. (**A**) Isotype distribution of protective mAbs. *n* = 222 mAbs from 67 studies; (**B**) Isotype distribution of indifferent mAbs. *n* = 252 mAbs from 30 studies; (**C**) Isotype distribution of disease-enhancing mAbs. *n* = 28 mAbs from 3 studies. Only mAbs with defined isotypes are counted.

Isotype function is important in designing antibody therapeutics and the alteration of isotype subclass can increase protective efficacy [122,123]. The reported percentage of IgG for protective and indifferent mAbs listed in Table 1 is 93% and 81%, respectively (Figure 3). There are about 5 fold more IgM for indifferent mAbs than protective mAbs. Interestingly, 44 of the 45 listed indifferent IgM are raised against either anthrax toxin or Shiga/Shiga-like toxin. A possibility for the relative scarcity of IgM comparing to IgG raised against toxins is that protein antigens trigger T-cell dependent responses resulting in B cell memory, and consequently, IgG becomes the primary isotype of an immunoglobulin to protein antigens. IgG1 is the predominant IgG subclass (Figure 3) [124], but the choice of immunization adjuvants can skew the immune system to produce more IgG of other subclasses [125,126]. Alternatively, the scarcity of IgM may have represented a bias for IgG in preserving hybridomas or lack of screening for IgM in the hybridoma development. IgM as a pentamer with large molecular weight is more difficult to purify, which makes it less attractive as therapeutics candidate. IgA plays important roles in mucosal immunity including the respiratory and gastrointestinal tracts, with primary functions of intracellular neutralization and immune exclusion [9]. However, protective IgA mAbs against toxins are rare that they are only reported in a ricin toxin study [82]. It should be noted that the nomenclature of IgG subclasses between human and mouse is different [127]. Although there is no exact correspondence in function between murine and human isotypes, human IgG1 is generally thought to be analogous to murine IgG2a or IgG2c depending on the mouse strains while human IgG2, IgG3, and IgG4 are considered analogous to murine IgG3, IgG2b, and IgG1, respectively. Although many murine mAbs have been used therapeutically in humans, advances in antibody engineering technologies allow the construction of chimeric and humanized antibodies which have the advantage of being less immunogenic and confer human constant region function [128].

## 3. Caveats of Protection Studies

Several caveats are observed from the mAb studies listed in Table 1, which may render the results difficult to interpret and compare. Some of them are discussed below, and the aims are to highlight the diversity of methods and experimental parameters in mAb works, and help guiding the design of future protection studies. 

### 3.1. Definition of Protection

It is sometimes difficult to define a mAb as protective, since protection may not be a clear cut phenomenon. It should also be noted that “neutralization” is a term that is generally reserved for interference with toxin action in an *in vitro* assays, while “protection” is a favorable outcome observed from *in vivo* studies. Pragmatically, protection can be defined as any gain of survival, viability, or fitness comparing to the control intoxicated condition. While any arbitrary percentage of increased survival can be defined as protection, the studies reviewed usually used a value ranging from 30% to 100%. The large range can then be divided into finer categories as “protective”, “slightly protective”, and “minimally protective”, indicating that the “protection” of mAbs is also relative to each other. Too little mAb cannot neutralize enough toxin to give protection, while too much mAb may result in prozone-like effects that reverse the protection observed with smaller amounts of mAb [129,130]. Consequently, most studies describe the mAb concentration being used to better standardize the protective efficacy of mAbs in the field, even though properties of an immunoglobulin such as binding affinity [131], avidity [132], degree of glycosylation [133], and effect of Fc region [123] are not as easily and commonly compared. 

### 3.2. Toxin and Bacterium Inoculum

The toxin concentration in an assay directly affects the readout of mAb efficacy. If too much toxin is used, the fine resolution of slightly protective mAbs may be lost, and vice versa. To detect the disease or toxin enhancing effect of a given mAb, a toxin concentration that results in relatively higher percentage of viability should be used. Typically, a toxin concentration that gives about 50% viability is a reasonable starting point to screen for both mAb-mediated protection and disease enhancement. To compare the efficacy between mAbs in different studies, it is important to consider the toxin and bacterial inoculum. For example, a fully protective mAb in one study may not have higher efficacy than a slightly protective mAb in another study that uses 1.5-fold more toxin in the system. In fact, the concentration of toxin can vary by as much as 5-fold in different studies [18,19]. However, this problem can be ameliorated by the inclusion of standardization that correlates toxin concentration and toxicity [18], since this allows comparison of results across studies. Another advantage of this measure is to minimize the uncertainty due to toxin shelf life and batch-to-batch difference. Side-by-side experiments can be conducted for the comparison of mAbs to reduce the effect of toxin quality on the neutralization assay. 

### 3.3. Screening Methods

Based on the need and availability, mAb studies employed different screening methods to test for mAb efficacy. Different methods can influence the efficacy readout, which makes the comparison of studies, even for the same toxin, challenging. For example, ADP-ribosylating, hemagglutinating, T-cell mitogenic, CHO cell-clustering, histamine-sensitizing, islet-activating, and leukocytosis-promoting activities have been used to define the efficacy of a mAb against pertussis toxin [134]. Similarly, mAb activity against anthrax toxin can be accessed by inhibition of anthrax toxin receptor and capillary morphogenesis protein (CMG2) binding, inhibition of pore formation, and inhibition of lethal toxin-mediated killing of macrophage-like cells [135]. Further complicating comparisons, a mAb may mediate protection in some but not all of the assays. The use of different cell lines can also complicate the comparisons of results from *in vitro* studies. Consequently, *in vivo* studies may be a better measure of mAb efficacy, and numerous studies have shown that mAbs reported to be neutralizing *in vitro* can fail to protect in animal models. 74.2% of neutralizing mAbs measured with *in vitro* assays also protect in animal model (Table 1). Yet, different species and strains have been used as animal models (Table 1), a common practice that contributes to the difficulty in comparing mAb across studies, since the genetic background affects the susceptibility to toxin [136]. The choice of animal model may be very different to the animal from which the antibody was made, with the examples of the testing of mAbs generated from chimpanzees in a rat model [62,70]. Furthermore, a single dose instead of a dose response is usually tested in most animal studies, an omission that could be very important if the former does not capture the optimum protection window for a particular toxin. In general, each of the screening assays plays a special role in revealing mAb efficacy. While *in vitro* studies can be used as the primary screening of potential mAb candidate and reveal the action of mechanism for mAb-mediated neutralization, the result of *in vivo* studies is still considered as the primary evidence for mAb efficacy. However, it is noteworthy that *in vitro* neutralization tests rely on interfering with mechanism that is part of the toxigenic process and that positive results may or may not translate into *in vivo* effects. Furthermore, mAbs whose function requires host factors such as cytokine, cell surface receptor, and complement to mediate a protective effect will fail to neutralize toxin or virus in a system where these components are lacking but could still be protective in animal model. 

### 3.4. Interactions of Multiple Monoclonal Antibodies

Since mAbs became available, most investigators have assumed that the effect of antibody can be defined by the immunoglobulin in isolation—mAbs are screened alone and characterized as protective, indifferent, and disease-enhancing. While this approach simplifies the study of antibody-mediated immunity, it neglects the fact that naturally-occurring antibody responses are complex polyclonal mixtures with diverse specificities and properties. The outcome of the interaction between multiple antibodies and toxin is complex and not easily predictable. In this regard, the combination of multiple protective mAbs that target different epitopes on the same antigen can synergize protection [43,137,138]. Mixing of mAbs that target anthrax toxin components, protective antigen and lethal factor, respectively, augments protection against toxin challenge [58]. A mixture of two non-protective mAbs rescued mice from Staphylococcal enterotoxin B-induced lethal shock by a mechanism whereby the binding of one mAb promotes structural change that allows the second one to neutralize the toxin [117]. The fact that antibody-mediated immunity is often protective, contrasts with the observation that most toxin-binding mAbs described in the literature are non-protective (Figure 1B). 

## 4. Conclusions

The properties of mAb that include high specificity, purity, and ability to enhance host immune system make immunoglobulins attractive candidates for the next generation of therapeutics. While mAb development is primarily focused on the candidates with the greatest medicinal potential, one should be cautious that the mAbs categorized as non-protective on single antibody screen, which comprise the majority of naturally-produced antibodies (Figure 1B), could be false-negative results. In other words, it is possible that those antibodies are biologically active when they function in combination with other antibodies. Combination testing could reveal new properties of these so called non-protective or indifferent antibodies with the caveat that evaluating antibodies in combinations would increase considerable cost and complexity to any screen for useful mAbs. However, the promising outcomes from mAbs cocktail against viral diseases and synergy between mAb and anti-infective drugs [9] suggest that this approach may unveil the potential of previously neglected mAbs. Perhaps the next generation technology will shift the focus of antibody development from single antibody screening to combination testing. Finally, continuous advancement of mAb engineering in the areas of mAb bispecificity, glycosylation, and Fc region modification will lay a solid foundation for the future mAb therapeutic era against toxin-mediated diseases. 
